# Phyllodes Tumor of the Breast Metastasizing to the Vulva

**DOI:** 10.1155/2015/589547

**Published:** 2015-04-15

**Authors:** Olusegun Kayode Ajenifuja, Nonna Kolomeyevskaya, Fadi Habib, Adekunle Odunsi, Shashikant Lele

**Affiliations:** ^1^Department of Gynaecological Oncology, Roswell Park Cancer Institute, Buffalo, NY 14263, USA; ^2^Department of Pathology and Laboratory Medicine, Roswell Park Cancer Institute, Buffalo, NY 14263, USA; ^3^Gynaecological Oncology Unit, Department of Obstetrics and Gynaecology, Obafemi Awolowo University Teaching Hospitals, Ile-Ife, Osun State 220005, Nigeria

## Abstract

Phyllodes tumors of the breast are rare breast tumors that resemble fibroadenoma. They are composed of two types of tissues: stromal and glandular tissues. Unlike fibroadenoma, they are commonly found in the third decade of life and they tend to grow more rapidly. Depending on the relative components of the cells and mitotic activity, they are classified into benign, borderline, and malignant. They are usually present as a lump in the breast. Phyllodes tumors are usually managed by wide excision. The excision should be wide enough to ensure a tumor-free margin. Recurrence rate is very high and most recurrences are usually local. Metastasis to the vulva has not been reported.

## 1. Introduction

Phyllodes tumors are rare forms of breast tumors constituting less than 1% of primary breast tumors [1]. They are composed of two types of breast tissues: connective and glandular elements [2]. They are subdivided into benign, borderline malignant, and malignant based on cellularity, stromal overgrowth, and degree of atypia. In most reported series less than 50% of phyllodes tumors are malignant. Though they can be found at all ages, they are, however, more commonly seen in the perimenopausal age group [3]. Clinical presentation of phyllodes tumors is indistinguishable from fibroadenoma [4], and diagnosis is only confirmed by detailed pathological examination of the entire tumor [5]. Treatment is by local wide excision but recurrence rate is said to be high. Local recurrences in other regions have been reported after treatment of the primary lesion but metastasis to the vulvar area is very rarely reported.

## 2. Case Presentation

We hereby describe an unusual case of a primary phyllodes tumor of the breast that metastasized to the vulva. The patient was a 55-year-old woman who was first diagnosed with phyllodes tumors of the breast in 05/2010. She was managed with wide excision of the tumor. One year later she had a recurrence in the right breast (04/2011) and three years later in the small bowel wall (05/2013). She was managed with excision of the tumors and she was commenced on chemotherapy.

The patient presented to our institution with an anterior left vulvar mass in 08/2013. She was managed with wide local excision.

All the previous excisions pathologies were requested from the outside institutions and reviewed at our center. The diagnosis of borderline phyllodes tumor of the breast was confirmed in the 2010 and 2011 breast excisions. The 2011 breast excision showed more stromal overgrowth and higher stroma/ducts ratio than the 2010 excision with more infiltrative and irregular borders. However, there was no necrosis or sarcomatoid transformation noted. The small bowel wall excision shows frank high grade sarcoma without any specific morphologic differentiation. CD117 was performed to exclude gastrointestinal stromal tumor (although CD117 can be positive in up to 35% of phyllodes tumor) and it was negative.

**Figure 1:**
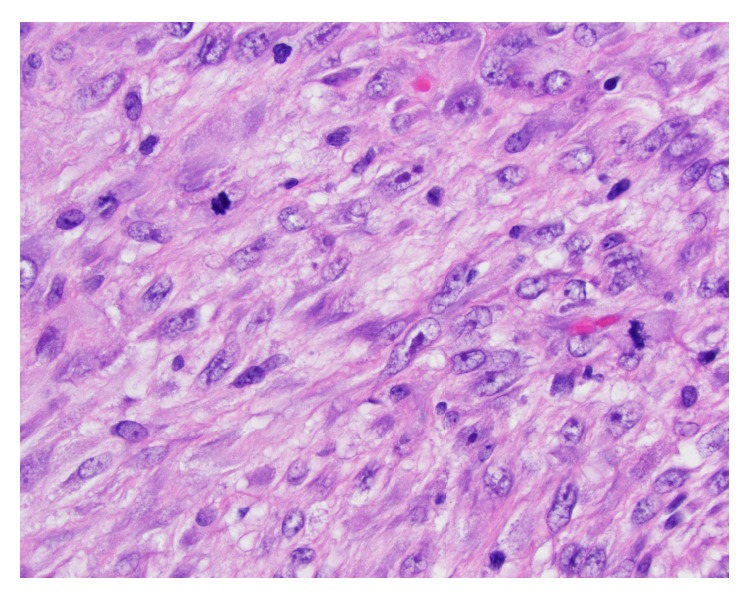
H&E staining of phyllodes tumor.

The gross examination of the vulvar mass shows a pink-tan lobulated soft tissue measuring 3.9 × 2.9 × 1.5 cm with overlying skin ellipse. The skin shows 2.6 × 1.8 cm white to tan ulcerated area. Sectioning reveals tan solid cut surface. The microscopic examination shows proliferation of pleomorphic neoplastic spindle cells with frequent mitotic figures and large atypical forms resembling the small bowel excision morphology. The current tumor is slightly less cellular than the metastatic sarcoma into small bowel which could be explained by the ongoing chemotherapy effect (Figure 1).

A large panel of immunohistochemical stains was performed to rule out other spindle cell neoplasms that can be seen in the anatomic site. The neoplastic tumor cells are positive for vimentin and negative for AE1/AE3, CK5/6, and CAM 5.2 immunostains. The tumor cells are negative for S100 (positive in melanoma), myogenin, MyoD1 (positive in spindle cell tumors of skeletal muscle differentiation), and SMA (positive in spindle cells tumor of smooth muscle differentiation). CD34 and CD117, which can be positive in phyllodes tumor up to 75% and 35%, respectively, were negative in the vulvar mass as well as the primary breast phyllodes tumor.

The overall morphologic features combined with the clinical history and immunohistochemical stains are diagnostic of high grade spindle cell sarcoma and consistent with the clinical history of phyllodes tumor from a breast primary.

## 3. Discussion

Metastatic tumors of the vulva are less common [6] and, in most cases, the primary lesions are usually located in the anogenital region. In a review by Chao and Sun of 78 metastatic tumors of the vulva, majority of cases (78%) originated from the cervix while less than 3% were from the breast [7]. One of the most common metastatic tumors from the breast to the vulva is intraductal carcinoma.

Metastatic breast tumors to the vulva are very rare and till date only 23 cases have been described in the English literature and are mainly Paget's disease and other glandular tumors.

The consensus among surgeons is that phyllodes tumors of the breast should be managed by wide excision [8]. Options of management range from wide excision to mastectomy. In one of the largest series so far on phyllodes tumors, Lin et al. suggested wide local excision with disease-free margin as the optimal management of these tumors. Recurrence rate could be as high as 10% [9]. In most cases recurrences are histologically similar to the primary tumor [10], but there is no agreement about the pattern of recurrence. One study is suggesting that rate of recurrence is racially determined with Asian women having more recurrences than their Caucasian counterparts [11]. Rowell and colleagues however found little correlation between the histological grade and pattern of recurrence [8]. Risk factors for recurrence depends on the histological grade of the tumor, positive resection margin, and over growth of stromal components. In some studies there was no correlation between tumor-free surgical margin and recurrence of benign phyllodes tumor [11, 12]. While for the borderline malignant type local excision was found to be associated with increased recurrence rate on univariate analysis [12].

The use of adjuvant therapy in the form of either chemotherapy of radiation therapy was advocated by Guillot et al, for recurrences [9]. This was found to lead to better local control but did not lead to increased survival [9].

Breast-like tumors of the vulva are rare. About 8 cases only of vulvar phyllodes tumors have been described [13]. Phyllodes tumors of the breast have been known to metastasize to other distant organs in the body but not to the vulvar area [14]; this case represents to the best of our knowledge the first reported case of a breast phyllodes tumor metastasizing to the vulvar area.
